# Antioxidant, Anti-Bacterial, and Congo Red Dye Degradation Activity of Ag_x_O-Decorated Mustard Oil-Derived rGO Nanocomposites

**DOI:** 10.3390/molecules27185950

**Published:** 2022-09-13

**Authors:** G. S. Lekshmi, Tamilselvi Ramasamy, Olha Bazaka, Igor Levchenko, Kateryna Bazaka, Raji Govindan, Mohandas Mandhakini

**Affiliations:** 1 International Centre for Research on Innovative Biobased Materials (ICRI-BioM), Lodz University of Technology, 90-924 Lodz, Poland; 2 Centre for Nanoscience and Technology, AC College of Technology, Anna University, Chennai 600025, India; 3 School of Science, College of Science, Engineering and Health, RMIT University, Melbourne, VIC 3000, Australia; 4 Plasma Sources and Application Center, National Institute of Education, Nanyang Technological University, Singapore 639798, Singapore; 5 Institute for Future Environments, Queensland University of Technology, Brisbane, QLD 4000, Australia; 6 School of Engineering, The Australian National University, Canberra, ACT 2601, Australia; 7 Department of Physics, Saveetha Institute of Medical and Technical Sciences (SIMATS), Saveetha University, Chennai 602105, India

**Keywords:** antioxidant, anti-bacterial, photocatalytic, nanocomposites, mustard oil

## Abstract

Scaling up the production of functional reduced graphene oxide (rGO) and its composites requires the use of low-cost, simple, and sustainable synthesis methods, and renewable feedstocks. In this study, silver oxide-decorated rGO (Ag_x_O−rGO) composites were prepared by open-air combustion of mustard oil, essential oil-containing cooking oil commercially produced from the seeds of *Brassica juncea*. Silver oxide (Ag_x_O) nanoparticles (NPs) were synthesized using *Coleus aromaticus* leaf extract as a reducing agent. Formation of mustard seed rGO and Ag_x_O NPs was confirmed by UV-visible characteristic peaks at 258 nm and 444 nm, respectively. rGO had a flake-like morphology and a crystalline structure, with Raman spectra showing clear D and G bands with an I_D_/I_G_ ratio of 0.992, confirming the fewer defects in the as-prepared mustard oil-derived rGO (M−rGO). The rGO-Ag_x_O composite showed a degradation efficiency of 81.9% with a rate constant *k*^−1^ of 0.9506 min^−1^ for the sodium salt of benzidinediazo-bis-1-naphthylamine-4-sulfonic acid (known as the azo dye Congo Red) in an aqueous solution under visible light irradiation. The composite also showed some antimicrobial activity against *Klebsilla pneomoniae*, *Escherichia* *coli*, and *Staphylococcus* *aureus* bacterial cells, with inhibition zones of ~15, 18, and 14 mm, respectively, for a concentration of 300 µg/mL. At 600 µg/mL concentration, the composite also showed moderate scavenging activity for 2,2-diphenyl-1-picrylhydrazyl of ~30.6%, with significantly lower activities measured for Ag_x_O (at ~18.1%) and rGO (~8%) when compared to control.

## 1. Introduction

Globally, water contaminated with infectious and chemical agents exposes individuals to otherwise preventable health risks, from cholera and diarrhoea to cancer. In addition to known risks from exposure to arsenic, fluoride or nitrate, there is a growing concern of the harmful effects of such contaminants as pesticides, pharmaceuticals, and dyes not only to human health but also to the health of the ecosystem, with significant negative socioeconomic consequences for the affected communities. Among industrial dyes, azo dyes and aromatic amines are considered to be profoundly cancer-causing [1,2]. In the textile industry, only about 80% of the used azo dyes end up being bound to the fiber, with the remaining 20% remaining in the effluent [3]. The presence of −N=N− bond is believed to be responsible for the cancer-causing activity and environmental persistence of azo dyes [4]. While the textile industry has been an essential segment of the economy for hundreds of years, the recent unprecedented growth in the demand for fast fashion and the use of synthetic dyes have substantially increased the impact of the industry on both human health and the environment [5]. At the same time, the management of industrial wastewater often remains inadequate, resulting in textile industries discharging harmful azo dyes directly into encompassing water bodies, affecting the availability of drinking water for hundreds of millions of people, often those already economically disadvantaged.

As is the case for many xenobiotics, once they enter the ecosystem, azo dyes tend to persist because their microbial degradation is hindered by their electron deficiency. The latter arises from the presence of electron-withdrawing groups, e.g., azo (−N=N−) and sulphonic (SO_3_^−^) moieties [6]. For such popular dyes as Congo Red (CR), which is widely used in textile, chemical and pharmaceutical industries [7], the degradation is further challenged by the presence of the benzidine-based anionic double-azo structure ([1-naphthalene sulfonic acid, 3,30-(4,40-biphenylenebis (azo)) bis (4-amino-)disodium salt], which renders it highly stable when exposed to water, light, elevated temperature and other chemicals [8]. The presence of an aromatic amine called benzidine in the CR dye also makes it carcinogenic and mutagenic. Nevertheless, it continues to be used in many countries.

Among the methods that have been tried for the removal of CR from effluent, adsorption, photocatalysis, Fenton process, and ozonation (as an advanced oxidation process) are considered most promising. One of the novel methods for CR removal is the graphene oxide-based technology. Graphene oxide flakes are two-dimensional materials with a large surface area, which makes it a promising material for numerous applications across electrical, electronic, biological and photocatalytic fields [9,10]. Conventional graphene synthesis methods are criticized for the release of toxic gases into the environment [11,12], which may reduce the environmental benefit gained from environmental remediation using thus-produced graphene. To eliminate the production of toxic gases and the use of strong reducing agents, environmentally friendly methods are being actively sought [13,14], especially those that could produce quality graphene at large scales needed for environmental applications. Among the available alternative methods, plasma-based technology [15,16] and physically similar but simpler flame synthesis [17,18,19,20] offer a favourable combination of processing simplicity and low cost of produced nanomaterials and metamaterials.

The photocatalytic activity of reduced oxides can be further enhanced by introducing other chemically reactive nanomaterials into the composites [21], e.g., silver nanoparticles [22]. In addition to having plasmonic properties, silver nanoparticles are highly toxic to a broad range of microbial cells, including pathogenic bacteria and fungi. Due to the favourable combination of the aforementioned properties, silver nanoparticles are promising for the development of novel anti-bacterial and antioxidant materials [23]. Silver nanoparticles synthesized using a plant-mediated environmentally friendly method show enhanced photocatalytic degradation ability towards Congo Red dye because of their suitable band gap (2.51 eV) [24]. However, the fast electron-hole recombination rate constrains its effective usage as a photocatalyst. Creating a composite of silver nanoparticles with graphene and its derivatives provides an effective means to prevent electron-hole recombination, while also significantly increasing the effective surface area where the chemical reactions can take place [25]. In addition to improved photocatalytic properties, these composites may retain the desired mechanical and chemical antimicrobial activity of graphene, as well as its biocompatibility with mammalian cells [26], providing a viable alternative for conventional anti-bacterial and antifungal agents [27,28].

In this study, we investigate the photocatalytic properties of silver oxide-decorated rGO (Ag_x_O−rGO) composites, where the rGO is synthesised via scalable, affordable, and simple open-air combustion of mustard oil, and silver oxide (Ag_x_O) nanoparticles are produced through simple mixing of a widely available and affordable silver precursor (AgNO_3_) with an aqueous extract from leaves of *Coleus aromaticus*, which functions as a phytochemical reducing and capping agent. Both mustard oil and leaf extracts are renewable, easily accessible feedstocks. This is in contrast with the traditional chemical production of reduced graphene oxide (rGO) that frequently involves the use of petrochemical precursors and specialised processing conditions that are expensive to maintain, and synthesis of silver nanoparticles, which is typically a slower and more complex process. After a series of washings with water and ethanol, the open-air combustion technique produced mustard oil-derived rGO with the yield of 1 mg of rGO for 0.1 g of oil. The anti-bacterial and antioxidant properties of the resultant composite are also investigated with the aim of developing a multi-functional material platform for wastewater management.

## 2. Experimental

### 2.1. Material Synthesis

All analytical-grade reagents, including AgNO_3,_ sodium salt of 3,3′-([1,1′-biphenyl]-4,4′-diyl)bis(4-aminonaphthalene-1-sulfonic acid) (Congo Red azo dye, CR) and ethanol (99%), were purchased from Sigma Aldrich Ltd., St. Louis, MO, USA. Commercial-grade mustard oil was purchased from a local market in Chennai and used as a precursor for this study without further modification. Leaves of *Coleus aromaticus* plants were obtained from the Anna University campus, Chennai, Tamilnadu, India.

Reduced graphene oxide (rGO) was prepared by burning mustard oil in a porcelain container using a surgical-grade cotton wick under open-air conditions, with the product collected on the surface of another thoroughly cleaned porcelain plate. The porcelain plate was kept at a distance of 0.15 m from the wick, with the distance value obtained as a result of optimisation. To produce each sample, 50 mg of mustard oil was used as the precursor (to yield 0.5 mg of rGO per sample) and the experiment was carried out for 2 h. The black-coloured product was obtained from the incomplete combustion of mustard oil precursor, where the carbon precursor undergoes decomposition and subsequent self-assembly into rGO flakes [29], involving a complex interplay of processes such as nucleation, surface growth, chain formation, diffusion, segregation, and precipitation. The product formed on the plate was washed with ethanol and water several times. The product was then sonicated for 2 h in water, transferred into a beaker, and dried at 80 °C.

**Figure 1:**
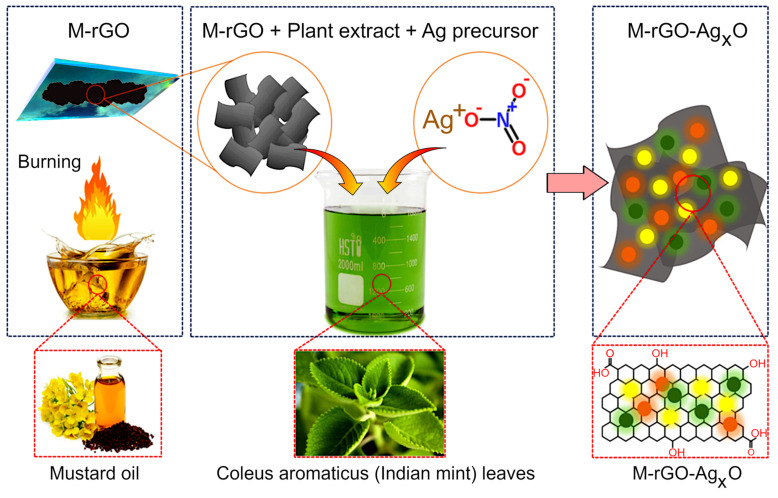
Schematic of the formation of M−rGO−Ag_x_O nanocomposite using simple open-air combustion of mustard oil. Silver oxide nanoparticles were produced by mixing AgNO_3_ silver precursor with the *Coleus aromaticus* leaf extract. The final nanocomposites were produced after 6 h stirring.

The leaf extract was prepared using an approach described previously in [30,31]. Approximately 10 g of freshly collected *Coleus aromaticus* leaves were washed thoroughly with de-ionized water and air dried at room temperature. Once dried, leaves were ground using a mixer grinder, and then extracted with distilled water by heating to 60 °C for 5 min, and then allowed to cool. Once cooled, the leaf extract was filtered and refrigerated at 4 °C for storage in an opaque bottle before further use. Silver oxide−rGO (Ag_x_O−rGO) composites were prepared via a single-step synthesis method. For that, 1 mM AgNO_3_ was combined with 90 mL of water and 20 mL of aqueous plant extract. The colour of the solution changed to turbid brown. Into that solution, 1 g of as-prepared rGO was added and kept under vigorous stirring for 6 h. Thus-obtained product was then dried and underwent structural and morphological analyses. The schematic representation of the process is shown in Figure 1.

### 2.2. Material Characterization

Structural and morphological analyses were conducted on rGO, Ag_x_O and Ag_x_O−rGO composite samples. For the structural analysis, X-ray diffraction analysis was performed using Rigaku Miniflex 600 with Cu K radiation of wavelength 0.154 nm and Raman spectra were collected using AGILTRON1 (QEB1920). Chemical characterisation was performed using Fourier Transform Infrared Spectroscopy (JASCO FT/IR 6600) operated in the attenuated total reflectance (ATR) mode over the range of 4000–500 cm^−1^. The UV-vis adsorption spectra were recorded using Shimadzu UV 800 spectrophotometer in the wavelength ranging from 200 to 800 nm. The surface morphology of samples was analysed using TESCAN-Vega scanning electron microscope, it being flaky in the case of rGO and particle-like in the case of Ag_x_O−rGO composites. Electrochemical impedance spectra (EIS) were collected using a Biologic VSP analyser under illumination (100 mWcm^−2^) at open circuit potential conditions over a frequency range of 10–10^6^ Hz.

The photocatalytic degradation of the dye was performed under visible light (at a wavelength of 460 nm). As previously mentioned, the degradation of CR dye via microbial or chemical pathways is challenging due to the presence of electron-withdrawing azo (−N=N−) and sulphonic (SO_3_^−^) chemical groups [6], as well as the benzidine-based anionic double-azo structure that gives CR dye its stability in the presence of water, light, elevated temperature and other chemicals [8]. For the experiment, 25 mg of as-synthesised rGO or Ag_x_O−rGO were dispersed in 100 mL of 10 ppm CR dye solution and stirred for 30 min in the dark. The samples were then kept under a 410 nm LED light, which was used as the visible light source. Every 60 min, a 5 mL sample was taken for UV-vis spectroscopic analysis to quantify the degradation efficiency. The degradation efficiency (%) was calculated using the equation: The degradation efficiency (%) = {(C_0_ − C))/C_0_} × 100, where C_0_ is the initial CR dye concentration and C is the equilibrium CR dye concentration, in mg/L.

The antimicrobial activity of thus-fabricated Ag_x_O, rGO and Ag_x_O−rGO composite samples was evaluated using a well-diffusion method. Gram-negative *Klebsiella* *pneumonia* and *Escherichia* *coli* and Gram-positive *Staphylococcus aureus* were used in the study. Specifically, the pure cultures of *Klebsiella pneumonia* MTCC 4030, *Escherichia coli* MTCC 1687 and *Staphylococcus aureus* MTCC 1687 are obtained from the Institute of Microbial Technology (IMTECH), Chandigarh, India. For each bacterial strain, a stock culture was created by using 20% glycerol nutrient broth (Oxoid, Basingstok, UK). The stocks were kept in the deep freezer at −80 °C. Before using these cells in an experiment, these cultures were refreshed by seeding the cells onto nutrient agar (Oxoid). Healthy colonies were transferred into flasks each containing 100 mL of nutrient broth and grown for 12–18 h at 37 °C with shaking at 120 rpm for the cells to enter the logarithmic growth stage. At this point, the haemocytometer was used to adjust the density of cells to OD_600_ = 0.3. Then, 100 µL of bacteria suspension was spread over agar and lysogeny broth media solidified in Petri dishes and allowed to grow to achieve confluency. The agar plates were prepared using 22 mL of agar medium per plate. Wells were punched in the agar, with each well receiving 100 µL of nanoparticle material suspended in deionised water at the concentration of 500 particles per mL. All experiments were conducted in triplicate, with all results expressed in terms of mean values and the corresponding standard deviations. SPSS v. 18.0 software package (IBM, Armonk, NY, USA) was used to determine statistical significance of the differences observed between different sample groups, with *t* tests used for statistical processing of data.

The radical scavenging activity of Ag_x_O, rGO, and Ag_x_O−rGO were measured using a 1, 1-diphenyl 2-picrylhydrazyl (DPPH) free-radical scavenging assay. Then, 1 mL of 0.1 mM DPPH solution in methanol was mixed with 1 mL of various concentrations (100–200 μg/mL) of samples. The mixture was then allowed to stand for 30 min incubation in the dark. Also, 1 mL of DPPH solution mixed with 1 mL of methanol was used as the control. The decrease in absorbance was measured at 517 nm. The percentage of inhibition was calculated using the following [32]:

(1) $${\%\mathrm{DPPH}}_{\mathrm{radical}\mathrm{inhibition}}=\frac{\mathrm{Control}-\mathrm{Sample}}{\mathrm{Control}}\times 100.$$

## 3. Results and Discussion

**Figure 2:**
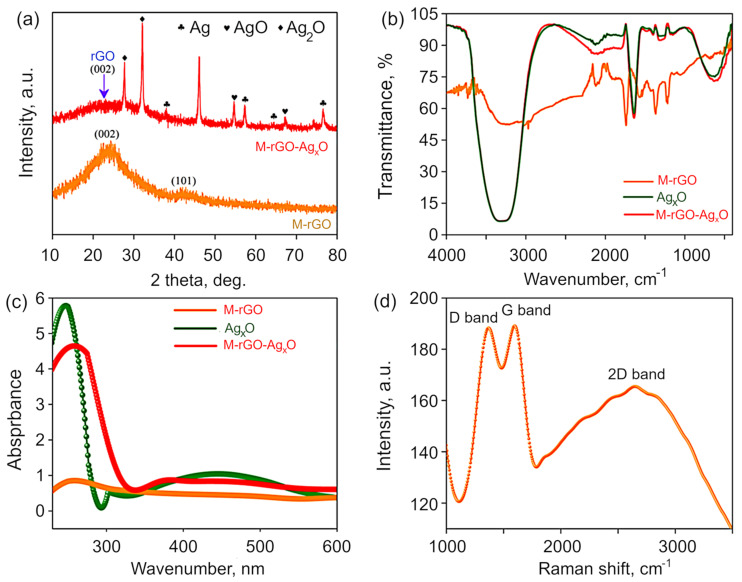
(**a**) Typical XRD patterns for M−rGO and M−rGO−Ag_x_O. Both spectra feature broad and intense peaks that indicates the presence of nanosized materials. (**b**) FTIR and (**c**) UV-vis spectroscopy spectra for M−rGO, Ag_x_O, and M−rGO−Ag_x_O, and (**d**) Raman spectrum of M−rGO.

The crystalline structure of the materials was confirmed by XRD, with the results of the XRD analysis for mustard seed oil-derived rGO and its Ag_x_O−rGO composite shown in Figure 2a. For both rGO and Ag_x_O−rGO composites, spectra show broad and intense peaks that indicate the presence of nanosized materials. The XRD peak at 2θ = ~24° is indexed to the (002) crystal plane and the diffraction peak at 2θ = 42°, corresponding to the (100) lattice plane of rGO. In graphite, the inter-layer distance (*d*) is in the range of 3.38–3.41 Å [33]. Here, *d* is calculated using Bragg’s equation as 0.381 nm for FRGO and 0.398 nm for M−rGO/Ag_x_O composite. The increase in the inter-layer distance in M−rGO/Ag_x_O composite is due to the presence of Ag oxides and other functional groups. In M−rGO/Ag_x_O composite, the data are matched with the JCPDS data for AgO (JCPDS No. 84-1108). In the M−rGO/Ag_x_O composite, there are main characteristic peaks obtained at 2θ of 39° (111), 43.5° (002), 66° (022) and 79° (113), which can be indexed as the cubic phase of Ag (JCPDS No. 04-0783). The spectra for the silver nanoparticles also show minor diffraction peaks at 32° (111), 37° (002), 54° (022), 65° (113) and 69° (222) planes along with the diffraction peaks for Ag and AgO (JCPDS No. 84-1108). Peaks at 27.7° (110) and 31.7° (111) correspond to Ag_2_O. Evidently, two unassigned peaks were observed; these could be attributed to the occurrence of some bioorganic compounds/protein(s) in the leaf extracts used for the synthesis of Ag NPS [34,35].

FTIR spectra presented in Figure 2b show that there is an intense peak at 3361 cm^−1^ attributed to OH-stretching vibration present in Ag_x_O and M−rGO/Ag_x_O, whereas the intensity of the peak for OH groups is reduced in M−rGO due to the hydrophobic nature of oil-derived rGO, as well as possibly due to the absence of AgOH in pure M−rGO. For M−rGO, the spectrum showed the stretching vibration of the C=O band between 1700–2200 cm^−1^, with a broad peak at 2108 cm^−1^ and a pronounced peak at 1799 cm^−1^ corresponding to C=O stretching carboxyl vibration, and other oxygen-containing C–O–C, C–O–H, and C–O bridges between 1463–1020 cm^−1^, e.g., the peak at 1365 cm^−1^ indicating the presence of a C-O bond. The peaks at 1744 cm^−1^, 1558 cm^−1^, 1043 cm^−1^ (epoxy), and 1212 cm^−1^ have lower magnitude when compared to the as-synthesized M−rGO due to the addition of plant extracts. The peak at 1617 cm^−1^ suggests the presence of C=C bond. The peak obtained at around 607 cm^−1^ indicates the presence of Ag–O bonds, which in turn confirms the presence of AgO in the composite structure [36].

The absorption peak at 255 nm in the UV-visible spectra (Figure 2c) is due to the lower amount of the oxygenated functional groups (such as carboxyl, epoxy, and carbonyl groups) in the material, which leads to the π-electron delocalization in as-produced M−rGO [37]. The peak at 451 nm gives the preliminary confirmation of the formation of Ag_x_O nanomaterials [38]. A new peak at 379 nm is observed after the composite is formed on the M−rGO surface. The peak at 379 nm in the absorption spectrum of the M−rGO/Ag_x_O nanocomposite is attributed to surface plasmons and the presence of silver nanomaterials [39].

Raman spectra (Figure 2d) were obtained to confirm the level of disorder and presence of defects in the crystal structure of the as-produced M−rGO. The intensity ratio between the D and G bands (I_D_/I_G_) provides an indication of the level of defects present in the composite structure. Here, the G band, which arises from the C−C bond stretching in all sp^2^ carbons, is present at 1368 cm^−1^ [40], and the D band is present at 1597 cm^−1^ which is frequently assigned by the breathing mode of k-point phonons of A_1g_ symmetry [41]. The I_D_/I_G_ value is calculated to be 0.992, which is higher than that previously reported for graphite structures because the sp^2^ domains in this material are smaller than that in the graphite [42]. The crystallite size (La = 4.4 (I_D_/I_G_)^−1^ is calculated as 0.443 nm, which is in good agreement with the XRD data.

**Figure 3:**
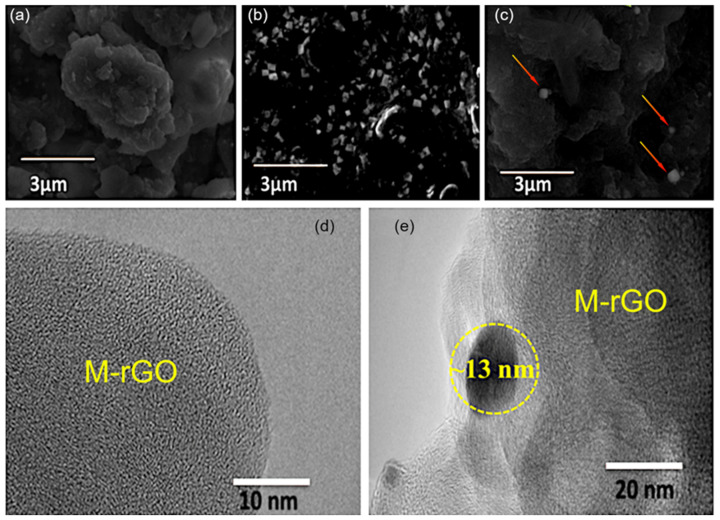
Representative SEM and TEM visualisation of the as-produced nanocomposites. (**a**) M−rGO, the M−rGO samples have a flake-like structure and are stacked; (**b**) Ag_x_O nanoparticles. Ag_x_O nanoparticles have a relatively uniform distribution in their shape, size and aggregation; (**c**) M−rGO─Ag_x_O nanocomposites, Arrows mark the Ag_x_O nanoparticles; TEM images of (**d**) M−rGO and (**e**) M−rGO─Ag_x_O nanocomposites. M−rGO comprises a number of nanosheets, forming the flake-like morphology.

The surface morphology of the as-prepared samples was characterised by SEM. From SEM images, it is seen that M−rGO samples have a flake-like structure and are stacked (Figure 3a). Figure 3b shows that Ag_x_O nanoparticles have a relatively uniform distribution in shape, size, and aggregation. From Figure 3c it is evident that in M−rGO/Ag_x_O nanocomposites, Ag_x_O nanoparticles are well distributed over the M−rGO flake surface. TEM images of M−rGO and M−rGO─Ag_x_O nanocomposite are shown in Figure 3d,e. TEM images confirm that the M−rGO comprises a number of nanosheets, forming the flake-like morphology. The Ag NPs are mostly spherical in size with a diameter of 13 nm uniformly distributed on the surface of M−rGO (Figure 3e).

**Figure 4:**
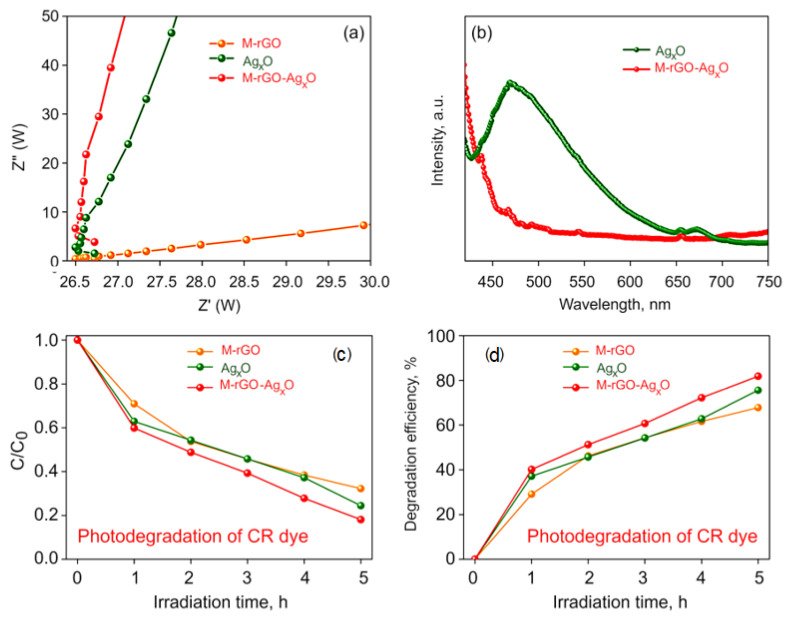
(**a**) EIS and (**b**) PL emission spectra for Ag_x_O and M−rGO/Ag_x_O nanomaterials. (**c**,**d**) Effect of contact time on photodegradation of CR dye using M−rGO, Ag_x_O and M−rGO/Ag_x_O hybrid.

The enhanced photocatalytic activity is obtained by extending the absorption edge of the material into the visible light region, increasing the electron-hole recombination rate. Electrochemical Impedance Spectroscopy (EIS) was used to analyse the charge separation and migration of charges. The Nyquist plots of M−rGO, Ag_x_O, and M−rGO─Ag_x_O nanocomposite are shown in Figure 4a. The Nyquist plot for the M−rGO─Ag_x_O nanocomposite shows a smaller arc radius compared to that for the M−rGO.

From these results, it is evident that the resistance is lower in M−rGO─Ag_x_O nanocomposite as compared to M−rGO and Ag_x_O. The higher conductivity obtained in M−rGO is due to the presence of aromatic rings in this material and the increased sp^2^ π conjugated areas [43,44]. Therefore, there will be a greater electron-hole transfer rate between the dye solution and the photocatalyst.

The electron-hole separation efficiency was investigated by photoluminescence (PL) analysis. Figure 4b shows the photoluminous intensity that is lower for M−rGO─Ag_x_O nanocomposites compared to Ag_x_O nanoparticles. This is due to the excitation of electrons from the valance band to the conduction band in Ag_x_O, which then migrate to the M−rGO sheets; this prevents the electron-hole recombination and thereby enhances the photocatalytic activity of the material [45,46].

The photocatalytic degradation of CR dye by M−rGO, Ag/AgO/Ag_2_O, and M−rGO− (Ag/AgO/Ag_2_O) hybrid photocatalysts is shown in Figure 4c,d at various time intervals. The photocatalytic degradation of CR dye in the presence of M−rGO, Ag/AgO/Ag_2_O, and M−rGO−(Ag/AgO/Ag_2_O) hybrid increases gradually with increasing irradiation time interval. The maximum degradation of CR dye in the presence of M−rGO, Ag/AgO/Ag_2_O and M−rGO−(Ag/AgO/Ag_2_O) hybrid is 67.77%, 75.55%, and 81.89% respectively, after 5 h irradiation. Photocatalytic degradation of Congo Red (CR) dye by M−rGO, Ag_x_O, and M−rGO−Ag_x_O nanocomposite photocatalysts is shown in Supplementary Figure S1.

The UV-visible spectra are shown at different time intervals. The photo-degradation of CR dye is found to increase as the pH decreases. The degradation efficiency is estimated to be 67.77% with the rate constant *k*^−1^ of 0.750 for M−rGO, 75.55% with the rate constant *k*^−1^ of 0.841 for Ag_x_O, and 81.89% with rate constant *k*^−1^ of 0.9506 for M−rGO−Ag_x_O.

The CR dye degradation efficiency and rate constants for M−rGO, Ag_x_O and M−rGO−Ag_x_O nanocomposite photocatalysts are listed in Table 1.

**Figure 5:**
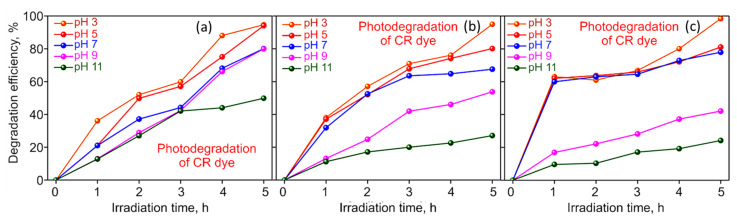
Effect of solution pH on photodegradation of CR dye using (**a**) M−rGO, (**b**) Ag_x_O, (**c**) M−rGO−Ag_x_O nanocomposite hybrids. The maximum equilibrium degradation capacity is obtained at pH 3 for all the three sample types.

The pH of the solution affects the adsorption and degradation dynamics since it modifies the ionization states of the binding groups of the dye and the photocatalyst [47]. The effect of pH of the dye solution on the rate of photodegradation of CR dye using M−rGO, Ag_x_O, and M−rGO−Ag_x_O nanocomposite photocatalysts are shown in Figure 5. The maximum equilibrium degradation capacity is obtained at pH 3 for all three sample types. At pH 3, degradation efficiency for M−rGO is 94.7%, for Ag_x_O is 95.0% and for M−rGO−Ag_x_O is 98.4%. From Figure 5 it is seen that the removal efficiency is at its lowest value at pH 11 for all the sample types. At pH 11, the degradation efficiency for M−rGO is 26.4%, Ag_x_O is 27.0% and for M−rGO−Ag_x_O is 51.3%. At lower solution pH, CR solution produces protonation of nitrogen atoms at amide and amines groups.

While increasing solution pH, the negative surface charge is developed on the photocatalyst surface which opposes the protonated amine groups and prevents contact between the surface of the photocatalyst and CR, leading to the reduction in the photocatalytic degradation of CR dyes [48]. In addition, lower pH may favor hydrogen bonding and the Van der Waals force of attraction in dye adsorption and degradation mechanisms [49].

**Figure 6:**
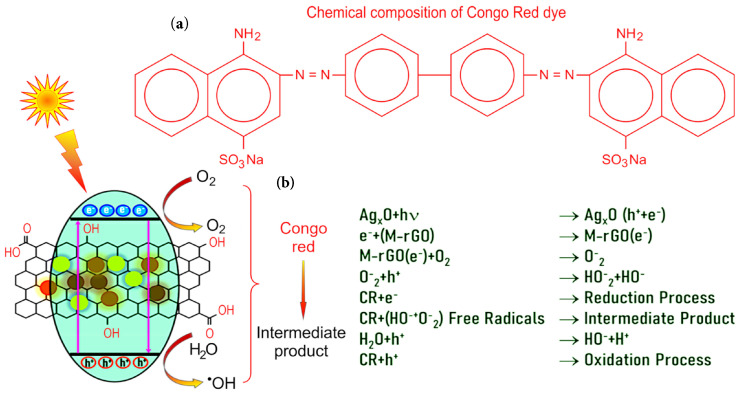
(**a**) Chemical composition of Congo Red dye. (**b**) Possible mechanisms for the degradation of Congo Red dye using M−rGO, Ag and Ag_2_O nanomaterials.

Electron-hole pairs are formed by the irradiation of sunlight on the surface of the photocatalyst. The main reasons behind the degradation of CR dye are O^−2^ ion and OH· radicals. The photoinduced electron reduces the molecular oxygen into ·O^−2^ ion. The highly reactive ·OH radical is produced in two ways: (i) the photogenerated holes h^+^ are captured by hydroxyl groups and produce highly reactive hydroxyl radicals (·OH); (ii) the photoinduced electrons reduce the adsorbed molecular oxygen O_2_ to the oxygen radical ·O^−2^, which is then oxidized by the hole to generate the HO_2_· radical (Figure 6).

**Figure 7:**
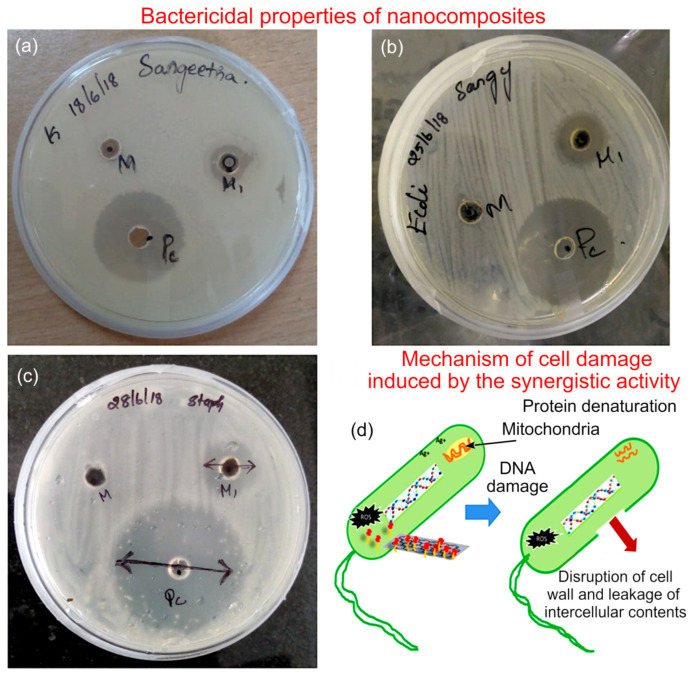
Antimicrobial efficacy of M−rGO−Ag_x_O against common bacterial species studied using the well-diffusion method. Representative plates of (**a**) *K. pneumonia*, (**b**) *E.* *coli*, and (**c**) *S.* *aureus*. (**d**) Proposed mechanism for the cell damage induced by the synergistic activity of M−rGO and Ag_x_O in the composite.

The bactericidal properties of M−rGO and M−rGO−Ag_x_O were also investigated against the three bacterial species, namely *Klebsiella pneumonia*, *Escherichia* *coli* and *Staphylococcus* *aureus*. Once inoculated with bacterial cells, plates were incubated until cells reached full confluence, at which point wells were made in the agar and 300 µg/m of either M−rGO, M−rGO−Ag_x_O, or Ag_x_O was placed into each well. Chloramphenicol, a broad-spectrum antibiotic that interferes with protein synthesis in bacteria, was used as the positive control. After 24 h, the zones of inhibition were measured, as shown in Figure 7, with the results of repeat experiments summarised in Table 2.

The M−rGO−Ag_x_O composite possesses a considerable broad-spectrum activity against the selected species of bacteria. In contrast, M−rGO has no discernible zone of inhibition. This may in part be attributed to the poor dispersion of M−rGO. The process of introducing silver nanoparticles to make the composite may result in a significantly improved dispersion of M−rGO particles, possibly resulting in a more pronounced anti-bacterial activity. Furthermore, the activity of reduced graphene oxide is synergistically enhanced by the activity of silver nanoparticles, the latter being a known anti-bacterial agent with a well-documented broad-spectrum activity [50].

Oxidative stress produced by M−rGO and Ag_x_O plays a major role in the anti-bacterial activity of the composite (Figure 7d). Graphene structures have a relatively high aspect ratio along the edges, and these sharp protruding features may contribute to the mechano-chemical destruction of bacteria. The features may induce physical damage to the membrane, whereas the presence of highly reactive species such as reactive oxygen species (ROS) produces oxidative stress which leads to cell damage or cell death. The presence of Ag_x_O also produces oxidative stress through the presence of free radicals [51]. Cytoplasm membrane rupture occurred by treatment with Ag ions, followed by its separation from the cell wall.

This results in the release of cellular contents and may lead to irreversible cell wall damage. The extraction of phospholipids from lipid membrane by M−rGO sheets is another cell destruction mechanism [52]. Silver ions Ag^+^ tend to associate with the R-SH groups present in the proteins [53]. That is, the extent of anti-bacterial activity of the Ag nanoparticles may be correlated with the fraction of R-SH groups present in the protein molecules or DNA. Hence, both the cell-penetrating properties of M−rGO sheets and the oxidative stress produced by both M−rGO and Ag_x_O are responsible for the destruction of bacteria [54].

**Figure 8:**
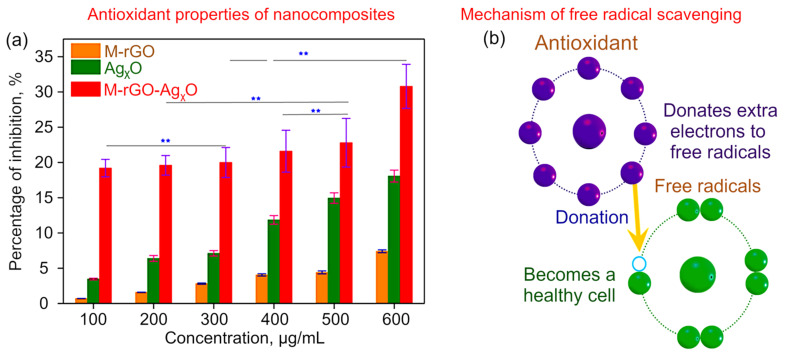
(**a**) Antioxidant properties of M−rGO and M−rGO−Ag_x_O nanocomposites demonstrated using DPPH free-radical scavenging assay. All values represent the mean ± SD. ** *p* < 0.05 (**b**) Pictorial representation of the mechanism of free-radical scavenging activity.

The production of free radicals can lead to numerous diseases like arthritis, skin disorder, tumours, and ulcers. It is necessary to stop the activity of free radicals by donating electrons from molecules and controlling these free radicals are recognized as antioxidants. DPPH was used for investigating the antioxidant activity based on the free radical scavenging activity of M−rGO, M−rGO−Ag_x_O, and Ag_x_O nanocomposites [55]. It is observed that the ability to scavenge DPPH radicals was increased with the increase in the concentration for all three samples (100–600 µg/mL), as shown in Figure 8. The DPPH scavenging activity is in the following order: M−rGO−Ag_x_O (30.6%) > Ag_x_O (18.1%) > M−rGO (8%). In the DPPH assay, antioxidant molecules with weak A−H bonds can donate hydrogen to the free radicals DPPH•, causing the discoloration of the solution [56]. From Figure 8a, it is evident that the free-radical scavenging activity is dose-dependent. Figure 8b illustrates the proposed mechanism of free-radical scavenging activity.

## 4. Conclusions

In this study, M−rGO−Ag_x_O composite photocatalyst was prepared using a facile flame synthesis method, and its efficiency for the removal of CR dye from aqueous solutions was studied under visible light conditions. Mustard oil was chosen as the precursor for M−rGO, as in addition to unsaturated, polyunsaturated, and saturated fats, it contains allyl isothiocyanate, a volatile organosulfur compound with the formula CH_2_CHCH_2_NCS. Ag_x_O nanoparticles were synthesized by the phytochemical method using *Coleus aromaticus* leaves extract to reduce the environmental impact of the synthesis. XRD and Raman scattering spectra clearly indicate the formation of M−rGO and Ag_x_O nanomaterials and the M−rGO−Ag_x_O nanocomposite. In addition to strong photocatalytic activity, M−rGO−Ag_x_O composite possesses good anti-bacterial activity against *Klebsiella pneumonia*, *Escherichia* *coli,* and *Staphylococcus* *aureus* bacteria, which is significantly greater than that of its components M−rGO and Ag_x_O. It should be mentioned that in this study, a detailed investigation of the effect of different nanoparticle concentrations on the bacterial survival was outside of the scope of this work, the aim of which was to demonstrate (i) the broad-spectrum antimicrobial potential of these types of nanocomposites against Gram-positive and Gram-negative bacteria, and (ii) the multi-functional nature of these nanocomposite platforms (i.e., antioxidant, anti-bacterial, and photocatalytic dye degradation activities). Similarly, M−rGO−Ag_x_O nanocomposites show better radical scavenging activity when compared to M−rGO and Ag_x_O. This study may offer a new strategy for the development of a low-cost environmentally friendly photocatalyst with better antioxidant and anti-bacterial activities.

## Figures and Tables

**Table 1:** CR dye degradation efficiency and rate constants for M−rGO, Ag_x_O and M−rGO−Ag_x_O nanocomposite photocatalysts.

Sample Type	Efficiency (%)	Rate Constant *(k*^−1^*)*
M−rGO	67.77	0.750
Ag_x_O	75.55	0.841
M−rGO−Ag_x_O	81.89	0.951

**Table 2:** The zones of inhibition for M−rGO and M−rGO−Ag_x_O and chloramphenicol.

	Zone of Inhibition ± SD, mm		
Active Agent	*K. pneumonia*	*E. coli*	*S. aureus*
Chloramphenicol	28 ± 2	28 ± 3	35 ± 4
M−rGO	NIL	NIL	NIL
M−rGO−Ag_x_O	15 ± 2	18 ± 3	14 ± 2

## Data Availability

Data is available on request.

## Supplementary Materials

The following supporting information can be downloaded at: https://www.mdpi.com/article/10.3390/molecules27185950/s1. Figure S1: Effect of contact time on the photodegradation of CR dye; Figure S2: Percentage of CR degradation at various solution pH.

## References

[1] DeMarini D.M., Carreón-Valencia T., Gwinn W.M., Hopf N.B., Sandy M.S., Bahadori T., Calaf G.M., Chen G., Conti A., Fritschi L. (2020). Carcinogenicity of some aromatic amines and related compounds. Lancet.

[2] Wang S., Hanna D., Sugamori K.S., Granta D.M. (2019). Primary aromatic amines and cancer: Novel mechanistic insights using 4-aminobiphenyl as a model carcinogen. Pharmacol. Therapeut..

[3] Ngo A.C.R., Tischler D. (2022). Microbial degradation of azo dyes: Approaches and prospects for a hazard-free conversion by microorganisms. Int. J. Environ. Res. Public Health.

[4] Al-Tohamy R., Ali S.S., Li F., Okasha K.M., Mahmoud Y.A.-G., Elsamahy T., Jiao H., Fu Y., Sun J. (2022). A critical review on the treatment of dye-containing wastewater: Ecotoxicological and health concerns of textile dyes and possible remediation approaches for environmental safety. Ecotoxicol. Environ. Saf..

[5] Sridharan R., Krishnaswamy V., Archana K.M., Rajagopal R., Kumar D.T., Doss C.G.P. (2021). Integrated approach on azo dyes degradation using laccase enzyme and Cul nanoparticle. SN Appl. Sci..

[6] Solís M., Solís A., Pérez H.I., Manjarrez N., Flores M. (2012). Microbial decolouration of azo dyes: A review. Proc. Biochem..

[7] Binupriya A.R., Sathishkumar M., Swaminathan K., Kuz C.S., Yun S.E. (2008). Comparative studies on removal of Congo red by native and modified mycelial pellets of Trametes versicolor in various reactor modes. Bioresour. Technol..

[8] González-Gutiérrez L.V., González-Alatorre G., Escamilla-Silva E.M. (2009). Proposed pathways for the reduction of a reactive azo dye in an anaerobic fixed bed reactor. World J. Microbiol. Biotechnol..

[9] Tamilselvi R., Ramesh M., Lekshmi G.S., Bazaka O., Levchenko I., Bazaka K., Mandhakini M. (2020). Graphene oxide-based supercapacitors from agricultural wastes: A step to mass production of highly efficient electrodes for electrical transportation systems. Renew. Energ..

[10] Tamilselvi R., Lekshmi G.S., Padmanathan N., Selvaraj V., Bazaka O., Levchenko I., Bazaka K., Mandhakini M. (2022). NiFe_2_O_4_/rGO nanocomposites produced by soft bubble assembly for energy storage and environmental remediation. Renew. Energ..

[11] Benzait Z., Chen P., Trabzon L. (2021). Enhanced synthesis method of graphene oxide. Nanoscale Adv..

[12] Hummers W.S., Offeman R.E. (1958). Preparation of Graphitic Oxide. J. Am. Chem. Soc..

[13] Romero A., Lavin-Lopez M.P., Sanchez-Silva L., Valverde J.L., Paton-Carrero A. (2018). Comparative study of different scalable routes to synthesize graphene oxide and reduced graphene oxide. Mater. Chem. Phys..

[14] Levchenko I., Mandhakini M., Prasad K., Bazaka O., Ivanova E.P., Jacob M.V., Baranov O., Riccardi C., Roman H.E., Xu S. (2022). Functional nanomaterials from waste and low-value natural products: A technological approach level. Adv. Mater. Technol..

[15] Piferi C., Bazaka K., D’Aversa D.L., Di Girolamo R., De Rosa C., Roman H.E., Riccardi C., Levchenko I. (2021). Hydrophilicity and hydrophobicity control of plasma-treated surfaces via fractal parameters. Adv. Mater. Interfaces.

[16] Piferi C., Carra C., Bazaka K., Roman H.E., Dell’Orto E.C., Morandi V., Levchenko I., Riccardi C. (2022). Controlled deposition of nanostructured hierarchical TiO_2_ thin films by low pressure supersonic plasma jets. Nanomaterials.

[17] Hong H., Xiong G., Dong Z., Kear B.H., Tse S.D. (2021). Open-atmosphere flame synthesis of monolayer graphene. Carbon.

[18] Zhang C., Tian B., Chong C.T., Ding B., Fan L., Chang X., Hochgreb S. (2022). Open-atmosphere flame synthesis of monolayer graphene. Combust. Flame.

[19] Kandasamy A., Ramasamy T., Samrin A., Narayanasamy P., Mohan R., Bazaka O., Levchenko I., Bazaka K., Mohandas M. (2020). Hierarchical doped gelatin-derived carbon aerogels: Three levels of porosity for advanced supercapacitors. Nanomaterials.

[20] Lekshmi G.S., Tamilselvi R., Geethalakshmi R., Kirupha S.D., Bazaka O., Levchenko I., Bazaka K., Mandhakini M. (2021). Biowaste valorization by conversion to nanokeratin-urea composite fertilizers for sustainable and controllable nutrient release. Carbon Lett..

[21] Carra C., Medvids A., Litvinas D., Ščajev P., Malinauskas T., Selskis A., Roman H.E., Bazaka K., Levchenko I., Riccardi C. (2022). Hierarchical carbon nanocone-silica metamaterials: Implications for white light photoluminescence. ACS Appl. Nano Mater..

[22] Banarjee P., Satapathy M., Mukhopahayay A., Das P. (2014). Leaf extract mediated green synthesis of silver nanoparticles from widely available Indian plants: Synthesis, characterization, antimicrobial property and toxicity analysis. Bioresour. Bioprocess.

[23] Ahmad S.A., Das S.S., Khatoon A., Ansari M.T., Afzal M., Hasnain M.S., Nayak A.K. (2020). Bactericidal activity of silver nanoparticles: A mechanistic review. Mater. Sci. Technol..

[24] Marimuthu S., Antonisamy A.J., Malayandi S., Rajendran S., Tsai P.-C., Pugazhendhi A., Ponnusamy V.K. (2020). Silver nanoparticles in dye effluent treatment: A review on synthesis, treatment methods, mechanisms, photocatalytic degradation, toxic effects and mitigation of toxicity. J. Photochem. Photobiol. B Biol..

[25] Hussain I., Singh N.B., Singh A., Singh H., Singh S.C. (2016). Green synthesis of nanoparticles and its potential application. Biotechnol. Lett..

[26] Kumari R.M., Thapa N., Gupta N., Kumar A., Nimesh S. (2016). Antibacterial and photocatalytic degradation efficacy of silver nanoparticles biosynthesized using Cordia dichotoma leaf extract. Adv. Nat. Sci. Nanosci. Nanotechnol..

[27] Maiti S., Kundu S., Ghosh D., Mondal S., Roy C.N., Saha A. (2016). Synthesis and spectral measurements of sulphonated graphene: Some anomalous observations. Phys. Chem. Chem. Phys..

[28] Kumar A., Aljumaili A., Bazaka O., Ivanova E.P., Levchenko I., Bazaka K., Jacob M. (2021). Functional nanomaterials, synergism and biomimicry for environmentally benign marine antifouling technology. Mater. Horiz..

[29] Yoo J.-Y., Jang E.-Y., Jeong S.-Y., Hwang D.-Y., Son H.-J. (2019). Bacterial indoleacetic acid-induced synthesis of colloidal Ag_2_O nanocrystals and their biological activities. Bioprocess Biosyst. Eng..

[30] Kumaran A., Karunakaran R.J. (2006). Antioxidant and free radical scavenging activity of an aqueous extract of Coleus aromaticus. Food Chem..

[31] Wadikar D.D., Patki P.E. (2016). Coleus aromaticus: A therapeutic herb with multiple potentials. J. Food Sci. Technol..

[32] Blois M.S. (1958). Antioxidant determinations by the use of a stable free radical. Nature.

[33] Kim J., Raj M.R., Lee G. (2021). High-defect-density graphite for superior-performance aluminum-ion batteries with ultra-fast charging and stable long life. Nano-Micro Lett..

[34] Sherin J., Kumar P.S., Karuthapandian S. (2021). Leaf extract arbitrated biogenic synthesis of silver nanospheres by a medicinal plant from the western ghats with enhanced antimicrobial property. Photochem.

[35] Khan M.Z.M., Tareq F.K., Hossen M.A., Roki M.N.A. (2018). Green synthesis and characterization of silver nanoparticles using Coriandrum sativum leaf extract. J. Eng. Sci. Technol..

[36] Haq S., Rehman W., Waseem M., Meynen V., Awan S.U., Saeed S., Iqbal N.J. (2018). Fabrication of pure and moxifloxacin functionalized silver oxide nanoparticles for photocatalytic and antimicrobial activity. Photochem. Photobiol. B Biol..

[37] Krishnamoorthy M., Veerapandian R., Mohan R., Kim S. (2012). Investigation of Raman and photoluminescence studies of reduced graphene oxide sheets. Appl. Phys. A Mater. Sci. Process.

[38] Perumbilavil S., Sankar P., Thankamani P.R., Philip R. (2015). White light Z-scan measurements of ultrafast optical nonlinearity in reduced graphene oxide nanosheets in the 400–700 nm region. Appl. Phys. Lett..

[39] Lee S.H., Jun B.-H. (2019). Silver nanoparticles: Synthesis and application for nanomedicine. Int. J. Mol. Sci..

[40] Akhavan O., Ghaderi E. (2010). Toxicity of graphene and graphene oxide nanowalls against bacteria. ACS Nano.

[41] Zhang X.P., Chen Y., Su Y., Wei G.Z. (2015). Recent advances in the fabrication and structure-specific applications of graphene-based inorganic hybrid membranes. Nanoscale.

[42] Liu S., Zeng T.H., Hofmann M., Burcombe E., Wei J., Jiang R., Kong J., Chen Y. (2011). Antibacterial activity of graphite, graphite oxide, graphene oxide, and reduced graphene oxide: Membrane and oxidative stress. ACS Nano.

[43] Huo J., Zeng H. (2016). Silver nanoparticles-sensitized cobalt complex for highly-efficient photocatalytic activity. Appl. Catal. B-Environ..

[44] Zhu W., Gao H., Zheng F., Huang T., Wu F., Wang H. (2019). Electrodeposition of graphene by cyclic voltammetry on nickel electrodes for microbial fuel cells applications. Int. J. Energy Res..

[45] Wang H., Zhang Q., Li J.-J., Zhang J.-Y., Liu Y., Zhou M., Zhang N., Fang Y.-Z., Ke Q. (2022). The covalent coordination-driven Bi_2_S_3_@NH_2_-MIL-125(Ti)-SH heterojunction with boosting photocatalytic CO_2_ reduction and dye degradation performance. J. Colloid Interface Sci..

[46] Padmanabhan N.T., Thomas N., Louis J., Mathew D.T., Ganguly P., John H., Pillai S.C. (2021). Graphene coupled TiO_2_ photocatalysts for environmental applications: A review. Chemosphere.

[47] Ghasemipour P., Fattahi M., Rasekh B., Yazdian F. (2020). Developing the ternary ZnO doped MoS_2_ nanostructures grafted on CNT and reduced graphene oxide (RGO) for photocatalytic degradation of aniline. Sci. Rep..

[48] Merrad S., Abbas M., Brahimi R., Trari M. (2022). Study of Congo Red removal from aqueous solution by using the deficient perovskite SrTiO_3-δ_ under solar light. J. Mol. Struct..

[49] Dutta S., Gupta B., Srivastava S.K., Gupta A.K. (2021). Recent advances on the removal of dyes from wastewater using various adsorbents: A critical review. Mater. Adv..

[50] Bhattacharyya A., Chattopadhyay R., Mitra S., Crowe S.E. (2014). Oxidative stress: An essential factor in the pathogenesis of gastrointestinal mucosal diseases. Physiol. Rev..

[51] Jung W.K., Koo H.C., Kim K.W., Shin S., Kim S.H., Park Y.H. (2008). Antibacterial activity and mechanism of action of the silver ion in Staphylococcus aureus and Escherichia coli. Appl. Environ. Microbiol..

[52] Tu Y., Lv M., Xiu P., Huynh T., Zhang M., Castelli M., Liu Z., Huang Q., Fan C., Fang H. (2013). Destructive extraction of phospholipids from Escherichia coli membranes by graphene nanosheets. Nat. Nanotechnol..

[53] Workentine M.L., Harrison J.J., Stenroos P.U., Ceri H., Turner R.J. (2007). Pseudomonas fluorescens’ view of the periodic table. Environ. Microbiol..

[54] Lekshmi G.S., Tamilselvi R., Geethalakshmi R., Kirupha S.D., Bazaka O., Levchenko I., Bazaka K., Mandhakini M. (2022). Multifunctional oil-produced reduced graphene oxide–Silver oxide composites with photocatalytic, antioxidant, and antibacterial activities. J. Colloid Interface Sci..

[55] Kumar S.R., Ashish J., Satish N. (2011). Momordica charantia Linn: A mini review. Int. J. Biomed. Res..

[56] Qiu Y., Wang Z., Owens A.C.E., Kulaots I., Chen Y., Kane A.B., Hurt R.H. (2014). Antioxidant chemistry of graphene-based materials and its role in oxidation protection technolog. Nanoscale.

