# Supplementary material for: Antioxidant, Anti-Bacterial, and Congo Red Dye Degradation Activity of AgxO-Decorated Mustard Oil-Derived rGO Nanocomposites
Source: Molecules. 2022 Sep 13;27(18):5950. doi: 10.3390/molecules27185950 (PMC9505018; doi:10.3390/molecules27185950)
Supplement: Supplementary file 1 [file molecules-27-05950-s001.zip › molecules-1845901-supplementary.pdf]

Supplementary Materials

Antioxidant, Anti-Bacterial, and Congo Red Dye Degradation Activity of Ag_xO-Decorated Mustard Oil-Derived rGO Nanocomposites

G. S. Lekshmi [1,2], R. Tamilselvi [2], Olha Bazaka [3], Igor Levchenko [4], Kateryna Bazaka [5,6,*], Raji Govindan [7] and M. Mandhakini [2,*]

[1] International Centre for Research on Innovative Biobased Materials (ICRI-BioM)—International Research Agenda, Lodz University of Technology, Lodz 90-924 Poland
[2] Centre for Nanoscience and Technology, AC College of Technology, Anna University, Chennai 600025, India
[3] School of Science, College of Science, Engineering and Health, RMIT University, Melbourne, VIC 3000, Australia
[4] Plasma Sources and Application Center, National Institute of Education, Nanyang Technological University, Singapore 639798, Singapore
[5] Institute for Future Environments, Queensland University of Technology, Brisbane, QLD 4000, Australia
[6] School of Engineering, The Australian National University, ACT 2601, Australia
[7] Department of Physics, Saveetha Institute of Medical and Technical Sciences (SIMATS), Saveetha University, Chennai 602105 India
* Correspondence: katia.bazaka@anu.edu.au (K.B.); mandhakini7@gmail.com (M.M.)

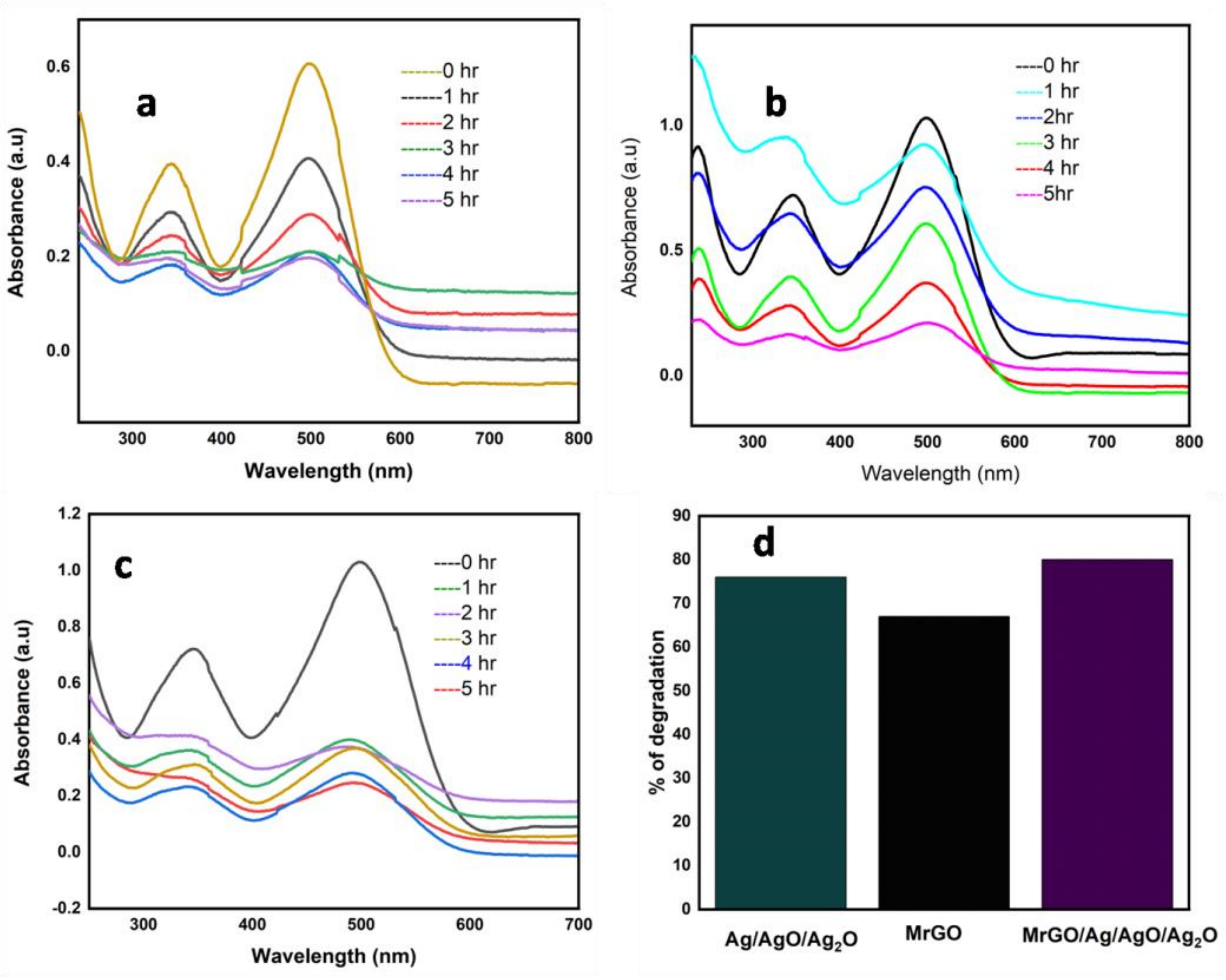

Figure S1. Effect of contact time on the photodegradation of CR dye using (a) M–rGO, (b) M–rGO–Ag_xO nanocomposite, (c) Ag_xO nanocomposites and (d) CR degradation efficiency after 5 h irradiation.

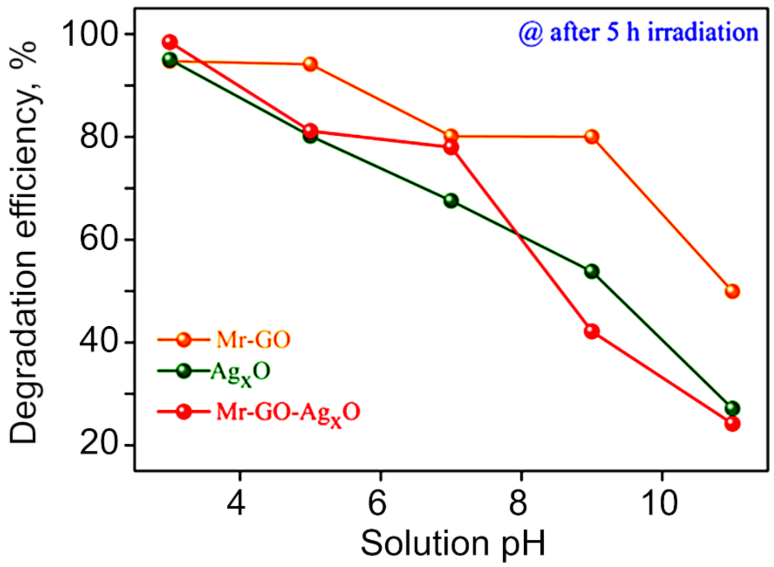

Figure S2. Percentage of CR degradation at various solution pH after 5 h irradiation.
